# Time-course gait pattern analysis in a rat model of foot drop induced by ventral root avulsion injury

**DOI:** 10.3389/fnhum.2022.972316

**Published:** 2022-12-19

**Authors:** Shu-Yen Chan, Chi-Wei Kuo, Tsai-Tsen Liao, Chih-Wei Peng, Tsung-Hsun Hsieh, Ming-Yuan Chang

**Affiliations:** ^1^Department of Medicine, College of Medicine, Taipei Medical University, Taipei, Taiwan; ^2^Graduate Institute of Medical Science, College of Medicine, Taipei Medical University, Taipei, Taiwan; ^3^School of Physical Therapy and Graduate Institute of Rehabilitation Science, Chang Gung University, Taoyuan, Taiwan; ^4^Cell Physiology and Molecular Image Research Center, Wan Fang Hospital, Taipei Medical University, Taipei, Taiwan; ^5^School of Biomedical Engineering, College of Biomedical Engineering, Taipei Medical University, Taipei, Taiwan; ^6^International Ph.D. Program in Biomedical Engineering, College of Biomedical Engineering, Taipei Medical University, Taipei, Taiwan; ^7^Neuroscience Research Center, Chang Gung Memorial Hospital, Taoyuan, Taiwan; ^8^Healthy Aging Research Center, Chang Gung University, Taoyuan, Taiwan; ^9^Division of Neurosurgery, Department of Surgery, Min-Sheng General Hospital, Taoyuan, Taiwan; ^10^Graduate Institute of Neural Regenerative Medicine, College of Medical Science and Technology, Taipei Medical University, Taipei, Taiwan; ^11^Discipline of Marketing, College of Management, Yuan Ze University, Taoyuan, Taiwan

**Keywords:** gait analysis, foot drop, ventral root avulsion, locomotor function, rats

## Abstract

Foot drop is a common clinical gait impairment characterized by the inability to raise the foot or toes during walking due to the weakness of the dorsiflexors of the foot. Lumbar spine disorders are common neurogenic causes of foot drop. The accurate prognosis and treatment protocols of foot drop are not well delineated in the scientific literature due to the heterogeneity of the underlying lumbar spine disorders, different severities, and distinct definitions of the disease. For translational purposes, the use of animal disease models could be the best way to investigate the pathogenesis of foot drop and help develop effective therapeutic strategies for foot drops. However, no relevant and reproducible foot drop animal models with a suitable gait analysis method were developed for the observation of foot drop symptoms. Therefore, the present study aimed to develop a ventral root avulsion (VRA)-induced foot drop rat model and record detailed time-course changes of gait pattern following L5, L6, or L5 + L6 VRA surgery. Our results suggested that L5 + L6 VRA rats exhibited changes in gait patterns, as compared to sham lesion rats, including a significant reduction of walking speed, step length, toe spread, and swing phase time, as well as an increased duration of the stance phase time. The ankle kinematic data exhibited that the ankle joint angle increased during the mid-swing stage, indicating a significant foot drop pattern during locomotion. Time-course observations displayed that these gait impairments occurred as early as the first-day post-lesion and gradually recovered 7–14 days post-injury. We conclude that the proposed foot drop rat model with a video-based gait analysis approach can precisely detect the foot drop pattern induced by VRA in rats, which can provide insight into the compensatory changes and recovery in gait patterns and might be useful for serving as a translational platform bridging human and animal studies for developing novel therapeutic strategies for foot drop.

## Introduction

Foot drop is a common gait impairment that is characterized by the inability to lift the forefoot and perform ankle dorsiflexion due to weakness of the dorsiflexors of the foot (Shaikh et al., 2017). The common compensations for a foot drop include steppage, circumduction gait, and a persistently abducted limb while walking. This, in turn, can lead to an unsafe antalgic gait, potentially resulting in falls (Nori and Stretanski, 2021 Dec). Foot drop also causes a significant deterioration of the physical and emotional aspects of the quality of life in a majority of patients (Aprile et al., 2005). The etiologies behind this presentation are varied and include myogenic or neurogenic factors such as dorsiflexor injuries, disc herniation, peripheral nerve injuries, stroke, neuropathies, drug toxicities, or diabetes (Bridwell et al., 1998).

In addition to myogenic and neurogenic processes, lumbar spine disorders (e.g., lumbar degenerative disease, intervertebral disc herniation, and spinal stenosis) are common neurogenic causes of foot drop (Stewart, 2008). Foot drop resulting from lumbar spine disorder is a special presentation of a severe motor deficit (Iizuka et al., 2009; Wang and Nataraj, 2014). Specifically, foot drop was reported in 8.1% of all inpatients with lumbar degenerative disease, including lumbar disk herniation, and lumbar spinal stenosis (Liu et al., 2013). The pathology is typically located at the L5 spinal root at the L4/5 spinal level, although the involvement of L5/S1 and multiple levels is also common (Wang and Nataraj, 2014; Suthar et al., 2015). However, the accurate prognosis of foot drop related to lumbar spine disorders is not well delineated in the scientific literature due to the heterogeneity of the underlying lumbar spine disorders, different follow-up times, and distinct definitions. Additionally, the recovery rate of foot drop in studies varies considerably (Emamhadi et al., 2016; Berger et al., 2021; Tanaka et al., 2021).

To further explore effective therapeutic strategies for improving the management of foot drops, it is essential to have a relevant and reproducible foot drop animal model. Such a model can be able to provide a more stable condition, standardized protocol, and controlled method of assessment to eliminate the discrepancies and further clarify the details of gait disturbance of foot drops and their treatment outcomes. However, the use of foot drop animal models for biomedical research is lacking or rarely properly addressed. Because one of the common causes of foot drop is nerve root damage in the lumbar spine, which could be obtained by ventral root avulsion (VRA). The VRA has been used as an animal model for nerve root injury for assessing the mechanisms of peripheral neuropathy and mimicking the clinical phenotype such as muscle weakness after peripheral motor nerve injury (Koliatsos et al., 1994; Oncu et al., 2006; Chang and Havton, 2016; Lovaglio et al., 2019). However, the foot drop animal model induced by VRA and the identification of the symptoms in this model are not yet developed.

For assessing foot drop symptoms *in vivo*, although gait analysis in rats has been applied in numerous neuroscience studies, the literature is sparse in terms of the time-course changes in motor behaviors or locomotion functions in the ventral root avulsion-induced foot drop rat model. Understanding the relationships between motor disturbances of foot drops after spinal root avulsion may provide substantial insight into the quantitative assessment of novel therapeutic strategies for patients. To analyze the detailed gait cycle for foot drop rats, it is essential to capture the spatiotemporal and kinematic parameters, such as detailed paw information and continuous joint kinematic data, to be recorded simultaneously during locomotion. In our previous studies, a video-based image processing system combined with footprint image acquisition was exploited for gait analysis in Parkinson’s disease, sciatic nerve injury, and Achilles tendon injury (Lee et al., 2012; Liang et al., 2012; Hsueh et al., 2014; Hsieh et al., 2021). Nevertheless, there is still no detailed gait analysis of foot drop animal models in current studies. Therefore, the present study aimed to develop a ventral root avulsion-induced foot drop rat model and establish a detailed analysis of the time-course changes in gait spatiotemporal and kinematic parameters for 3 weeks following the unilateral lumbar root avulsion procedure.

## Materials and Methods

### Animals

Twenty-five male Sprague Dawley rats (weight 350–400 g) were obtained from the BioLASCO Taiwan Co., Ltd. After surgery, the rats were housed in standard cages for 4 weeks at a temperature of 25°C and a humidity of 50%, with a 12 h light/dark cycle. Rats were free to move around the cage, and were not restricted in their food supply. All animal procedures were approved by the guidelines and rules of the Institutional Animal Care and Use Committee at Chang Gung University.

### Surgical procedures of completed ventral root avulsion injury for inducing foot drop in rats

**Figure 1:**
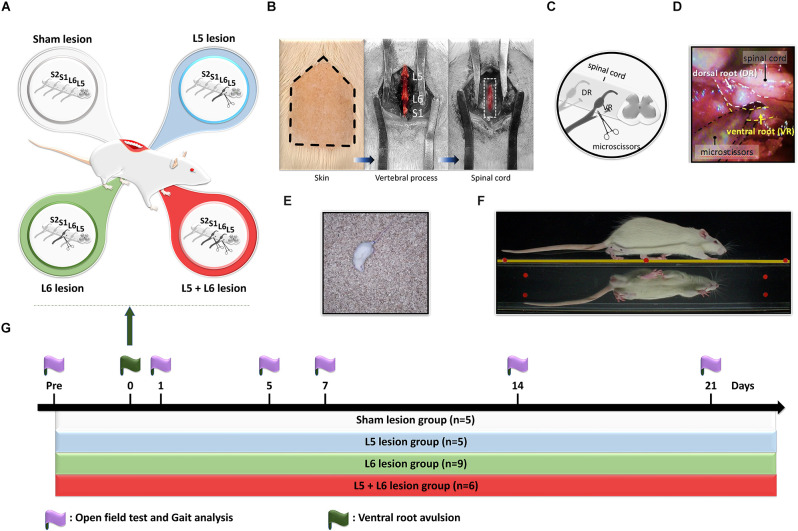
Design of the study for time-course changes of gait and locomotion functions in ventral root avulsion induced foot drop rat model. **(A)** Twenty-five rats were divided into four groups, including sham lesions (white), L5 lesions (blue), L6 lesions (green), and L5 + 6 lesions (red). **(B)** The hair of the lower back region of a rat was shaved for right L5 and L6 ventral root avulsion procedures. Paraspinous muscles and tendons were removed to expose transverse processes of the L5, L6, and S1. Then the L5 and L6 hemilaminectomy was conducted for better exposure of the spinal cord (dash line square) and the right ventral roots. **(C)** A schematic representation of the surgical ventral root avulsion model indicating the rhizotomy of the L5-L6 ventral roots. **(D)** The ventral root was grabbed with fine forceps and transected 3–4 mm proximal to the dorsal root ganglion with microscissors. **(E)** The locomotor function was assessed by the open field test in rats. **(F)** The gait pattern was evaluated by the transparent walking track. **(G)** Experimental design for time-course behavioral assays following ventral root avulsion in rats. Two behavioral tests, including an open field test and gait analysis, were performed pre-procedure and on days 1, 5, 7, 14, and 21 post-avulsions to investigate the changes in the gait parameters over time.

For induction of different severity of foot drop in a rat model, the separate completed ventral root avulsion of L5, L6, or L5 + L6 was performed in each group, respectively (Figure 1A). For completed ventral root avulsion, procedures were performed as described in a previous study with some modifications (Li et al., 2003; Chew et al., 2013; Chang and Havton, 2016). The surgical procedures were conducted using aseptic manipulation under an operating microscope. The adult male rats were anesthetized with a mixture of Rompun (0.1 ml/kg; Bayer, Germany) and Zoletil 50 (0.9 ml/kg; Virbac, France). The back of each rat was shaved, sterilized, and fixed on a plastic plate. To induce foot drops in rats, a midline skin incision (3–4 cm) was made in the region of the L4 to S1 vertebrae, and the paraspinous muscles and tendons were removed over the L5 vertebral dorsal process (Havton, 2012). The sacrum was identified first, and L5 and L6 right hemilaminectomy were subsequently performed, as shown in Figures 1B–D. Then, the transverse processes of the L5 and L6 vertebrae were removed to obtain good exposure to the L5 and L6 spinal roots. The L6 right side root sheath was exposed with scissors as it entered the vertebrate column. The right L6 ventral root and dorsal root were identified visually under a surgical microscope (Leica M300, Leica Microsystems, Switzerland) but the corresponding L5 dorsal root ganglion (DRG) was not exposed. The right L6 ventral root was identified as it is usually located at the most lateral side of the spinal canal just beneath the dorsal root. The ventral root was grabbed with fine forceps and transected 3–4 mm proximal to the DRG. Approximately 1 mm of the root was dissected (Sheth et al., 2002; Jeon et al., 2011). Great effort was taken to avoid any damage to the L6 dorsal roots and its DRG. After confirming homeostasis, the muscles and skins were closed in layers with 4–0 silk sutures. Following the operation, the rats were kept warm until they awakened and then returned to their respective cages. In the L5 + L6 group, the right L5 ventral root avulsion was performed after the above procedures of the right L6 ventral root. The rats in the sham-operated group received an incision, followed by removal of the L5 and L6 transverse processes and wound closure without damaging the spinal cord and roots.

### Locomotor activity

The open-field test was employed to measure general locomotor activity (Silva et al., 2016; Su et al., 2018; Feng et al., 2020). In this test, each rat was monitored in an open field black Plexiglas arena (60 × 60 × 100 cm in dimension) by a video camera (C930e, Logitech, USA). Three parameters including the total travel distance, the movement time, and the immobile time of each animal were recorded within a 10 min testing period (Feng et al., 2020). Each trial was recorded and analyzed by using a tracking system (Smart 3.0, Panlab, Harvard Apparatus, Barcelona, Spain). In order to avoid odor interference in the test response, the testing area was cleaned with 75% ethanol thoroughly between each testing period for all rats (Figure 1E).

### Spatiotemporal gait analysis

To identify the time-course changes in foot drop symptoms in the foot drop rat model, a video-based gait analysis system integrated with a walking track was applied to obtain the spatiotemporal parameters of gait in this study. The procedure to quantify and record the gait pattern in rats was described previously (Hsieh et al., 2011; Lee et al., 2012; Liang et al., 2012; Feng et al., 2020). Briefly, the walking system was constructed of transparent Plexiglas (80 cm × 6 cm × 12 cm) with a 45-degree tilting mirror underneath the walking platform, which would reflect an image of the rat’s paws for convenient evaluation with a digital camera. Additionally, a 20 cm × 6 cm × 2 cm cage with mobile gates was placed at each terminal end of the walkway. LED lights illuminated it from the superior-anterior and inferior-anterior directions. For image capture, a digital camera (PX-100, JVC, Japan) was positioned 1 m in front of and at the same level as the walkway to simultaneously capture the direct lateral view of the rat from the walkway and the bottom view from the mirror (Figure 1F). To obtain the precise spatial and temporal gait parameters, the digital camera was set at 60 frames per second (fps) at a resolution of 2,048 × 1,536 pixels. Following calibration, the spatial and temporal resolutions were 0.24 mm and 16.67 ms, respectively. Before the experiment, all rats were allowed to adapt to the walking track by walking freely on the walkway for 20 min before formal recording‥ Each trial was repeated until five or six satisfactory walks of at least four steps without pauses were captured. Only the hindlimb stepping patterns were analyzed in our current video-based gait analysis system.

For spatial gait analysis, the data were processed by MATLAB software (MathWorks, version 9.6., R2019a, Natick, Massachusetts USA) to identify the sequential footprints and kinematic parameters. Five parameters were included in our present study: stride length, step length, print length, toe spread, and intermediary toe spread. In terms of temporal gait analysis, the stance phase time, swing phase time, double support time, and walking speed were obtained. In addition, the kinematic parameters of the hindlimbs were measured. Three landmarks, namely, the central part of the shank, the lateral malleolus, and the fifth metatarsal head, were identified by MATLAB software to determine the range of motion (ROM) during the swing and stance phases of the gait cycle from the observations of the toe contact and toe-off in each step cycle. The ROM of the ankle joint at the four gait events, including initial contact (the time point when the foot or toes touch the ground), mid-stance (the time point when the contralateral swinging limb is opposite the stance limb), pre-swing (the time point when the foot is pushed and lifted off of the ground) and mid-swing (the time point when the swinging limb is opposite the contralateral stance limb) was manually identified from three landmark positions on the knee, ankle, and 5th metatarsal head (Lee et al., 2012; Liang et al., 2012). All the gait parameters including spatiotemporal gait indices and ROMs of the ankle joint at the four gait events were averaged for at least 10 footsteps or joint angles.

### Experimental design

Ventral root avulsion was undertaken in 20 rats (L5 avulsion, *n* = 5, L6 avulsion, *n* = 9 and L5 + L6 avulsion, *n* = 6). Animals were randomly divided into four groups (i.e., L5 ventral root avulsion, L6 ventral root avulsion, L5 + L6 ventral root avulsion, and sham-operated). Following ventral root avulsion, no mortality was noted. All rats, including sham lesion rats and ventral root avulsion rats, were assessed by the open field locomotor activity test, spatiotemporal gait, and kinematic ROM analysis on the pre-avulsion day and 1, 5, 7, 14, and 21 days post-ventral root avulsion surgery (Figure 1G).

### Statistical analysis

SPSS 25.0 package software (IBM SPSS Statistics for Windows, Armonk, NY, USA) was used to perform data analysis. The significance level was set at *p* < 0.05. For all behavior measurements, a mixed ANOVA was performed to test the between-subjects factor (L5, L6, L5 + L6 ventral root avulsion vs. sham group) and time (pretest vs. posttest) as the within-subject. When ANOVA showed that the main effect of the group was significant, *post-hoc* Fisher’s Least Significant Difference (LSD) tests were further utilized to compare the groups at each time point. Additionally, separate one-way ANOVAs as well as *post-hoc* Bonferroni tests were performed to compare the behavioral data at different time points as necessary. Data are expressed as the mean and standard error (mean ± SE).

## Results

### Kinematic gait pattern

**Figure 2:**
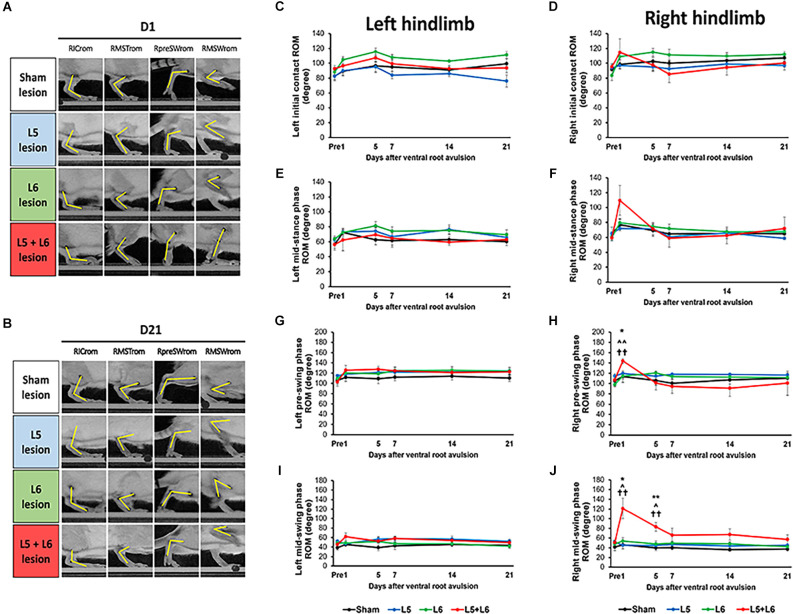
Characteristics of stepping of rats (as indicated by kinematic joint angle). Ankle joint angles were obtained from the sagittal view and determined from the landmarks on the knee, ankle, and 5th metatarsal head. Photographs of each group of rats in right initial contact ROM (RICrom), right mid-stance ROM (RMSTrom), right pre-swing ROM (RpreSWrom), and right mid-swing ROM (RMSWrom) on the 1st day **(A)** and 21st day **(B)** post-lesion. Time-course changes in ROM of the ankle joint in the left **(C)** and right **(D)** hindlimb at initial contact, left **(E)** and right **(F)** hindlimb at mid-stance phase, left **(G)** and right **(H)** hindlimb at pre-swing phase, left **(I)** and right **(J)** hindlimb at mid-swing phase. Note the significant increase in ROM in both the pre- and mid-swing phases after L5 + L6 ventral root avulsion at Day 1 post-lesion. Error bar = SEM. *p < 0.05, **p < 0.01 significant differences are shown between the sham and L5 + L6 groups. ^∧^*p* < 0.05, ^∧∧^*p* < 0.01 indicate significant *post hoc* Fisher’s LSD differences when compared between L5 lesions and L5 + L6 lesions. ^††^*p* < 0.01 indicates significant *post hoc* differences between the L6 lesion and the L5 + L6 lesion groups.

The detailed video-based gait analysis test was assessed in the sham group (*n* = 5), single L5 (*n* = 5), single L6 (*n* = 9), and L5 + L6 (*n* = 6) avulsion groups at baseline and 1, 5, 7, 14, and 21 days after the ventral root avulsion. Figure 2 illustrates the examples of the joint angles of the affected right ankle at four specific gait events (initial contact, mid-stance, pre-swing, and mid-swing) captured from a rat from each group on the 1st day and 21st days post-lesion (Figures 2A,B). In the unaffected hindlimb, no significant differences were found between the two groups in four specific gait events at each time point (all *p* > 0.05; Figures 2C,E,G,I). In the affected hindlimb (right side), a mixed ANOVA showed no significant difference was found on TIME × Group interaction at initial contact, mid-stance (all *p* > 0.05). There were no significant time-course changes in ROM among the four groups in the right initial contact phase and right mid-stance phase (*p* > 0.05; Figures 2D,F). However, a mixed ANOVA revealed significant time × group interaction at the pre-swing phase (*F*_15,100_ = 2.231, *p* = 0.01) and mid-swing phase (*F*_15,100_ = 4.760, *p* < 0.001; Figures 2H,J). Moreover, the subsequent *post-hoc* LSD test in the mid-swing phase between the L5 + L6 groups and single L5 or single L6 or sham group on post Day 1 was significant (all *p* < 0.05). The *post-hoc* tests in the mid-swing phase revealed that the ankle ROM in L5 + L6 reached a significantly higher degree than the sham group on the 1st day (*p* = 0.011) and 5th day (*p* = 0.003) post-avulsion but not at 2 weeks (*p* = 0.053) or 3 weeks post-avulsion (*p* = 0.09). There was also a significant difference between the L5 + L6 group and the L5 (*p* = 0.023) and L6 groups (*p* = 0.003) on the 1st and 5th days.

### Spatiotemporal gait analysis

**Figure 3:**
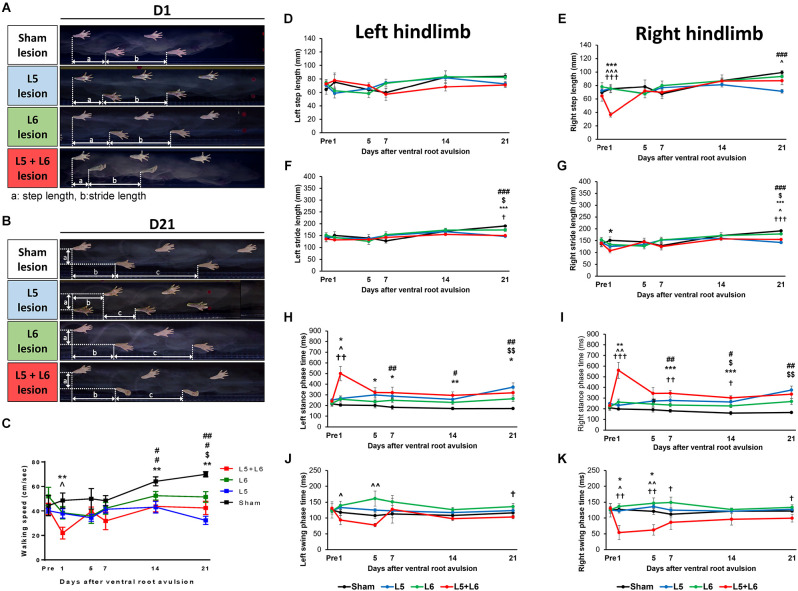
Tracking of hindlimb footprints during locomotion in four different groups on Day 1 and Day 21 post-avulsion. The heels as the reference point for measuring the distance (a) step length and (b) stride length were obtained from the bottom view. Photographs of each group of stepping footprints of rats on the 1st day and 21st day post-lesion in gait analysis **(A,B)**. The time-course changes in the walking speed **(C)** and left and right step and stride length of four groups over 21 days of observation **(D–G)**. The time-course changes in the stance phase and swing phase duration in the left and right hindlimb **(H–K)**. Note that the footprints in L5 + L6 ventral root avulsion rats clearly showed smaller step lengths, especially on the right (affected) side, however, the sham group rats displayed a comparatively consistent step length. *p < 0.05, **p < 0.01, ***p < 0.001 significant differences are shown between the sham and L5 + L6 groups. ^∧^*p* < 0.05, ^∧∧^*p* < 0.01, ^∧∧∧^*p* < 0.001 indicate significant *post hoc* Fisher’s LSD differences when compared between L5 lesions and L5 + L6 lesions. ^†^*p* < 0.05, ^††^*p* < 0.01, ^†††^*p* < 0.001 indicate significant *post hoc* differences between the L6 lesion and the L5 + L6 lesion groups. ^$^*p* < 0.05, ^$$^*p* < 0.01, indicates significant *post hoc* Fisher’s LSD differences when compared between the sham lesion group and L6 ventral root avulsion. ^#^*p* < 0.05, ^##^*p* < 0.01, ^###^*p* < 0.001 indicate significant *post-hoc* differences when compared between the sham lesion group and L5 ventral root avulsion.

Figure 3 demonstrates a representative series of footprint images captured from each group on the 1st day (Figure 3A) and 21st day post-lesion (Figure 3B). The ventral view of the footprints showed that L5 + L6 VRA rats walked with markedly shorter steps and strides than the pre-lesion rats and the L5 or L6 VRA rats. The pre-lesion and sham groups exhibited a relatively consistent step length.

Figure 3C indicates that there was a significant difference in the walking speed between groups since the walking speed of the post-avulsion rats was severely decreased. A significant difference was observed across time (*F*_5,100_ = 4.321, *p* = 0.001) and a significant group effect (*F*_3,20_ = 7.480, *p* = 0.002) was also observed for the changes in walking speed. No significant difference in the time × group interaction was found (*F*_15,100_ = 1.480, *p* = 0.127). Interestingly, a *post-hoc* group comparison showed that L5 + L6 ventral root avulsion rats walked significantly slower than the sham group on the 1st, 7th, and 21st days post-lesion (all *p* < 0.01).

Figures 3D–G illustrates the time-course changes in the left and right step and stride length in the four groups. In the unaffected hindlimb, a mixed ANOVA showed no significant effects of time × group interaction in the left step and stride length (*p* > 0.05; Figures 3D,F). However, a mixed ANOVA revealed a significant effect of time × group interaction (*F*_15,100_ = 2.669, *p* = 0.002) in the right step length. The *post-hoc* test showed that a significant difference was found between the L5 + L6 VRA group and the other groups on Day1 post-lesion (*p* < 0.05). A similar pattern can be seen for stride length (Figures 3F–G).

Regarding temporal gait parameters, post ventral root avulsion, Figures 3H–K reveals the precise duration of the left and right stance and swing phase. A significantly longer stance phase in L5 + L6 compared to the sham group persisted for 21 days during our entire measurement period. For the left stance phase time, a mixed ANOVA revealed significant effects of time × group interaction (*F*_15,100_ = 3.213, *p* < 0.001; Figure 3H). For the right stance phase time, a mixed ANOVA in the right stance phase time over 21 days revealed a significant effect in time (*F*_5,100_ = 6.654, *p* < 0.001) as well as a time × group interaction (*F*_15,100_ = 6.231, *p* < 0.001) and groups (*F*_(3,20)_ = 7.932, *p* < 0.001). *Post-hoc* LSD test analysis showed a significant difference between L5 + L6 and the other two groups on the 1st day post-lesion in left and right stance phase time (all *p* < 0.05; Figure 3I).

For the swing phase time, no significant main effect and interaction were found in the left hindlimb (Figure 3J). However, for the right hindlimb, a mixed ANOVA revealed a significant decrease in duration in groups (*F*_3,20_ = 6.950, *p* = 0.002) as well as a significant effect of the time × group interaction (*F*_15,100_ = 2.070, *p* = 0.017). More specifically, the swing phase duration in L5 + L6 dropped dramatically on day 1 post-lesion. *Post-hoc* comparisons between groups demonstrated that significant differences were found in swing phase duration in the L5 + L6 group compared to the sham group, single L5, and single L6 VRA group in the first 5 days post-VRA (*p* < 0.05; Figure 3K).

### Stepping footprint analysis

**Figure 4:**
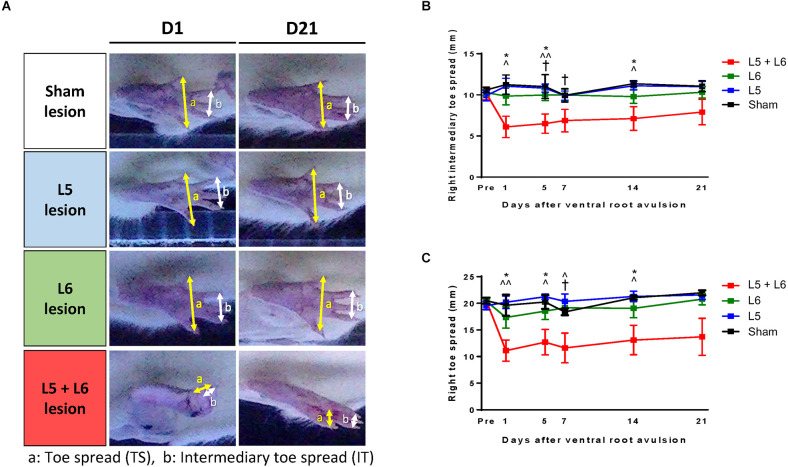
Characteristics of the stepping footprint during locomotion of post-lesion rats **(A)**. (a) Toe spread (distance between first and fifth toes) was obtained from the bottom view of the walkway (b) Intermediary toe spread (distance between the second and fourth toes) was obtained from the bottom view of the walkway. The time-course changes in the right intermediary toe spread **(B)** and right toe spread **(C)** of each group. Note that the footprints in L5 + L6 ventral root avulsion rats clearly demonstrated a shorter toe spread (approximately 11.61 mm ± 2.79 mm) and an intermediary toe spread (approximately 6.88 mm ± 1.37 mm) compared to the other three groups and this was maintained for the full 21 days of our observation period. *p < 0.05, **p < 0.01, ***p < 0.001 significant differences are shown between the sham and L5 + L6 groups. ^∧^*p* < 0.05, ^∧∧^*p* < 0.01, ^∧∧∧^*p* < 0.001 indicate significant *post hoc* Fisher’s LSD differences when compared between L5 lesions and L5 + L6 lesions. ^†^*p* < 0.05, ^††^*p* < 0.01, ^†††^*p* < 0.001 indicate significant *post hoc* differences between the L6 lesion and the L5 + L6 lesion groups.

The toe spread and intermediary toe spread of the post-lesion animals were also severely decreased, as shown in Figure 4A. In addition, in Figures 4B,C, it clearly showed that the right toe spread in L5 + L6 ventral root avulsion significantly shorter than the sham group, single L5 or single L6 group (*p* < 0.05) from post-avulsion day1 to day14 days. A mixed ANOVA of the toe spread over the 21 days revealed a significant main effect in time (*F*_5,100_ = 5.088, *p* < 0.001), groups (*F*_3,20_ = 4.977, *p* = 0.01) as well as a time × group interaction (*F*_15,100_ = 2.457, *p* = 0.004). Moreover, *post-hoc* analysis showed significance between L5 + L6 and the sham group from Day 1–14 days post-lesion (*p* < 0.05). A similar pattern can also be seen in the intermediary toe spread parameter. The distance between the second and fourth toes (intermediary toe spread) in the L5 + L6 group exhibited a dramatic drop compared to the other groups on Day 1 post-avulsion and maintained a stable low status until the end of our 21-day time course measurement period.

**Figure 5:**
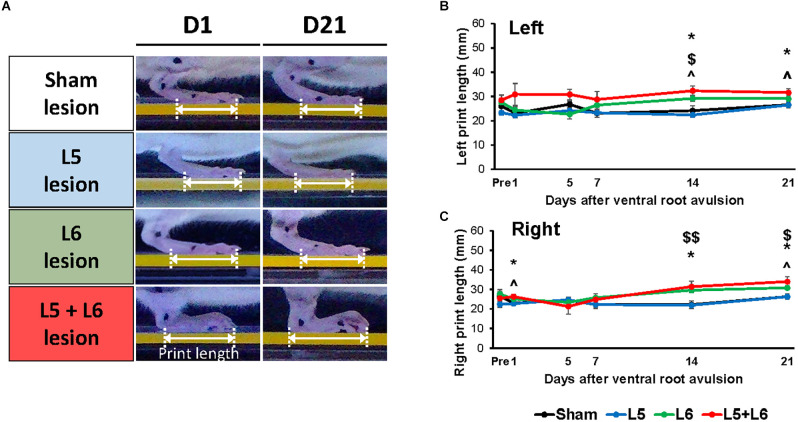
Characteristics of the stepping footprint during locomotion of post-lesion rats **(A)**. The time-course changes of print length of the sham and VRA lesion rats in the walkway locomotion test during the 21 days **(B,C)**. Levels of significance: *p < 0.05, indicated a significant difference between the sham and L5 + L6 groups; ^∧^*p* < 0.05 was used to mark the post hoc LSD difference between L5 + L6 lesion vs. L5 lesion; ^$^*p* < 0.05, ^$$^*p* < 0.01 were used to mark the post hoc LSD difference between L6 lesion vs. sham lesion group. Data are expressed as the mean ± SEM.

For the right print length, Figure 5A illustrates the time-course changes in the print length of the right foot among the four groups. Significant main effects were observed for time (*F*_5,100_ = 5.763, *p* < 0.001) and group (*F*_3,20_ = 4.318, *p* = 0.017). No significant main effect in the time × group interaction (*F*_15,100_ = 1.603, *p* = 0.086) was found. The *post-hoc* comparisons between groups in left and right foot are shown in Figures 5B and 5C, respectively. The L5 + L6 ventral root avulsion group had a longer print length than the sham group and single L5 group on post-avulsion Day 1, Day 14, and Day 21, respectively (all *p* < 0.05).

### Locomotor activity analysis

**Figure 6:**
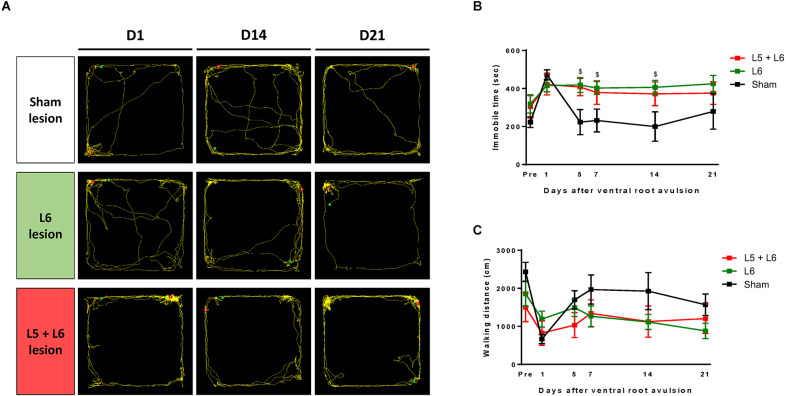
The effect of ventral root avulsion lesions in the time-course analysis of the locomotor activity of rats with an open field test. A representative trajectory plot of rats 1, 14, and 21 days after ventral root injury surgery **(A)**. Time-course change among groups in immobile time **(B)** and walking distance **(C)**. Values are expressed as the mean ± SEM. ^$^*p* < 0.05, a significant difference between the L6 and sham groups.

In the open field test, the overall distance traveled and immobility time was calculated to investigate the general locomotor activity between the sham group and the single L5, single L6, and L5 + L6 groups following lumbar ventral root avulsion (Figure 6A). A mixed ANOVA indicated a significant main effect of time (*F*_5,75_ = 5.263, *p* < 0.001), time group interaction (*F*_10,75_ = 2.329, *p* = 0.019), and group (*F*_2,15_ = 6.383, *p* = 0.01) on immobile duration during these 21-day time-course observations. When compared with the sham group, subsequent *post-hoc* tests showed that immobile duration (Figure 6B) time was significantly increased on the 5th day post-ventral root avulsion lesion in the L6 (*p* = 0.018) and L5 + L6 (*p* = 0.041) groups compared to the sham group and was maintained at a high level for up to 14 days after avulsion in both the L6 and L5 + L6 groups (all *p* < 0.05). However, even though we can see the trend following root avulsion, walking distance in the L5 and L5 + L6 groups both decreased compared to baseline and the sham group. For walking distance, a mixed ANOVA indicated a significant main effect of time (*F*_5,75_ = 6.094, *p* < 0.001) but not a time × group interaction (*F*_10,75_ = 1.845, *p* = 0.067) or group (*F*_2,15_ = 2.085, *p* = 0.159; Figure 6C).

## Discussion

This study evaluated and analyzed time-course changes in motor behaviors, including spatiotemporal gait patterns, ankle kinematic ROM, and open field locomotor activity, in a foot drop rat model for 3 weeks following ventral root avulsion. After ventral root avulsion surgery, this animal model successfully demonstrated the foot drop phenomenon, such as weakness of dorsiflexion of the foot, leading to increased ankle ROM during the mid-swing phase, which aligned with previous studies (Wang et al., 2008; Sharif Bidabadi et al., 2019). Compared to the sham and single L5 or single L6 avulsion groups, L5 + L6 ventral avulsion rats exhibited a gradual decrease in step/stride length, toe spreading, and walking speed. The gait cycles of the stance and swing phases were also affected.

The proposed video-based approach overcomes the limitations of conventional article footprint measurement (Hsieh et al., 2011; Liang et al., 2012). It provides a precise and broad spectrum of gait indices over video-based gait analysis systems, including detailed paw information, spatiotemporal and kinematic changes covering abnormal gait patterns in foot drop rats. In summary, the most important finding of our study is the establishment of a successful rat model mimicking foot drop gait characteristics due to lumbar radiculopathy in humans; both L5 + L6 ventral root avulsion is necessary. Single L5 or single L6 avulsion does not efficiently exhibit the characteristics of foot drop based on our gait and ROM parameters. With regard to the methodologies for evaluating the foot drop, several assessment tools were developed in human studies. For example, it is common to apply force plates, motion capture systems, or electromyography (EMG) to quantify the symptoms and compensatory behaviors in patients with foot drop (Geboers et al., 2002; Knarr et al., 2013; Wiszomirska et al., 2017; Blazkiewicz and Wit, 2019). However, animal studies for determining the symptoms are related rare due to no relevant and reproducible foot drop animal models and less suitable gait analysis methods were developed. To the best of our knowledge, this is the first research to develop a ventral root avulsion-induced foot drop rat model and show the detailed time-course changes of gait patterns in the foot drop rat model. In future studies, to more detailed identify the compensatory and behavioral changes, it is suggested to apply other methodologies such as force plates, motion capture systems, or EMG for getting more comprehensive behavior information serving as a translational platform bridging human and animal studies.

With regard to kinematic parameters, for the ROM of the ankle during four different phases, the mid-swing phase is the most critical phase defining foot drop (Simonsen, 2014; Seel et al., 2016). Foot drop is a gait impairment that is characterized by the inability to raise the foot during walking due to weakness of the dorsiflexors of the foot. During the swing phase, the foot is raised by the anterior tibialis muscle. In our study, the ankle ROM during the mid-swing phase in the L5 + L6 ventral root avulsion group was significantly higher than that in the sham, single L5, and single L6 groups. Therefore, the L5 + L6 ventral root avulsion rats align with the definition of drop foot.

Our present study clearly showed that single L5 or single L6 ventral root avulsion does not result in obvious foot drop symptoms in rats for the following reasons. First, most of the major muscles are innervated by nerves that generally extend from multiple roots or branches; for instance, the brachial and lumbar plexuses. It is reasonable that we need to damage at least two lumbar ventral roots to see clear foot drop symptoms in our animal model. Second, the ankle joint exhibits two degrees of freedom. Thus, the activation of both the tibialis anterior and fibularis longus muscles must be balanced to generate a straight (physiological) foot lift (Seel et al., 2016). Regarding nerve innervation, the tibialis anterior and fibularis longus are innervated by the deep peroneal nerve (from the L4, L5 roots) and the superficial peroneal nerve (from the L5-S2 roots), respectively. They are branches of the common fibular nerve (from the L4-S2 root branch; Drake et al., 2014). Rats show similar muscle branches and nerve innervation as humans. However, there are six pairs of lumbar spinal nerves in rats instead of five in humans (Ding et al., 2017). Based on previous studies, the sciatic nerve in rats originates predominantly from spinal segments L4–L6, which further distally give rise to the tibial, peroneal, and sural nerves and a cutaneous branch (Schmalbruch, 1986; Prodanov and Feirabend, 2007). Regarding the initial contact and mid-stance phase of the gait cycle, there was no significant difference in the degree of ankle joint movements between the sham and ventral root avulsion groups in our study, consistent with other clinical research (Baker, 2018).

Our detailed temporal-spatial gait analysis demonstrated that L5 + L6 ventral root avulsion led to a significant rise in the stance and reduction in the swing phases of the gait cycle in the first week post-avulsion. This could be explained by the fact that to increase balance and stability, ventral root avulsion rats tend to remain longer in the stance phase during locomotion. We can see that at the end of the swing phase, toe strikes before heel strikes resulted in the early ending of the swing phase time because of weakened ankle dorsiflexors, which further resulted in a shorter swing phase in the gait cycle. Furthermore, when compared to the sham groups, we determined that lumbar ventral root avulsion induced foot drop rats walked significantly slower and had to make shorter steps to move the same distance as healthy ones, which can be explained as necessary to increase balance and stability during locomotion. In additon when compared with the unaffected hindlimb, it is found that the ankle ROM during under swing phase was greatly influenced in the right hindlimb (lesioned side) of L5 + L6 VRA rats whereas the left hindlimb (unlesioned side) was not affected. These asymmetries were also found in the step length and swing phase time in the right hindlimb (Figure 3). The asymmetric pattern could be attributed to the compensatory pattern for increasing force and muscle activity during locomotion, which has been observed in patients with drop-foot (Knarr et al., 2013; Blazkiewicz and Wit, 2019).

Regarding the toe spreading parameter, our 21-day post-lesion data revealed a persistent, significant decrease in right toe spreading and right intermediate toe spreading in the L5 + L6 group compared to the sham group. The lower toe spread level shown in the L5 + L6 group could be influenced by abductor muscles in the foot. The abductor hallucis, abductor digiti minimi, and dorsal interossei are the three main muscles controlling foot spreading (Drake et al., 2014) and they are innervated by the tibial nerve and deep peroneal nerve, which also branch from the sciatic nerve. Thus, the rats with drop feet could not spread the hind paws due to L5 and L6 root avulsion. In terms of print length, due to damage to the deep peroneal nerve, the extensor muscles of the foot were not able to function (Drake et al., 2014). Contraction of the paws during the gait cycle was noticed in our study. Thus, the print length decreased in the first 5 days. However, it increased 1 week after root avulsion, which may be due to the weakened tibialis anterior and lateral malleolus of rats dropping down toward the ground, causing an increased length or footprint.

The time-course observation of gait patterns and behavior tests helped confirm the behavioral motor compensation after ventral root-induced foot drop and quantify the detailed gait and ROM parameters during the time changes. Across the 3 weeks of behavior test analysis, we discovered that immobile time and walking distance gradually recovered in the open field test after 2 weeks. Regarding ROM, ankle degree in the L5 + L6 group showed a constant higher degree for 21 days, whereas there was no significant difference after 7 days. Similarly, the swing phase time decreased dramatically in the L5 + L6 group on the 1st day post-avulsion but gradually reached a relatively stable plateau after a week. In this study, compared to the sham groups, we also found that all three lesion groups showed a significantly longer stance phase until 21 days following ventral root avulsion. Regarding toe spreading parameters, the L5 + L6 group showed a stable, significantly lower value than the other groups for 21 days. Moreover, the changes in fast recovery of gait parameters after ventral root avulsion could be related to the great recovery capacity of the axons in the peripheral nerve system. According to other studies, after avulsion injuries in rats, massive death of motor neurons occurs (Wu, 1993; Wu and Li, 1993). It is known that such proximal lesions induce the loss of approximately 80% of axotomized motor neurons during the first 2 weeks after injury (Koliatsos et al., 1994; Piehl et al., 1995). The capacity of the peripheral nervous system to regenerate lost axons after nerve injuries is well recognized (Bendszus and Koltzenburg, 2001; Menorca et al., 2013; Gordon and Borschel, 2017). In addition, compared with human beings, rodent axons have a shorter distance to travel before reaching the target tissue, so injury leaves the distal nerve stump denervated for a shorter period and there is a fast recovery (Scheib and Höke, 2013). This finding can further explain the fast time-course gait recovery of the foot drop rats in our study.

Following L5 + L6 ventral root avulsion, an open field behavior test was performed for locomotor activity evaluation. Compared to dynamic gait analysis, the open field test helps evaluate general locomotor activity levels after long-term physical training, anxiety, and willingness to explore in rats, but it is not a highly sensitive tool for assessing foot drop (Denenberg, 1969; Dishman et al., 1996; Ennaceur, 2014). Both walking distance and immobile duration time were affected in the L6 and L5 + L6 groups. In comparison to the pre-lesion baseline and sham groups, both L6 and L5 + L6 showed a significant increase in immobile time after 5 days of ventral root avulsion in our study. For walking distance, a substantial decrease in walking distance was observed 1-week post-lesion. Nevertheless, there was no significant difference between the groups. This enabled us to understand that following ventral root avulsion, rats tend to decrease their exploratory behaviors, which may be explained by the inability to walk normally due to foot drop symptoms or pain caused by nerve damage and tissue swelling. However, further research is still needed to clarify the detailed mechanisms of this action.

## Conclusion

This study utilized a video-based gait investigation device to confirm several spatiotemporal and kinematic indices of lateral hindlimbs and footprints during overground walking in lumbar ventral root-induced foot drop rats. The proposed analysis techniques are reliable and reproducible assessment tools to determine gait impairment and compensatory gait patterns following ventral root avulsion. A significant increase in the degree of the ankle joint was noticed in the mid-swing phase of the foot drop rat model, showing consistency with human drop foot subjects. The time-course changes in gait impairment started as soon as day 1 post-lesion and progressively recovered, reaching a plateau state approximately 2 weeks following ventral root avulsion. The video-based methodology and the experimental data illuminate new insight into the progressive changes affecting rodent gait patterns and suggest that the L5 + L6 ventral root avulsion rat model can be used as an animal model for human foot drop resulting diseases. Future researchers may use the presented methodology and data for an enhanced understanding of the general mechanisms of foot drop and for the development of novel treatment protocols for functional recovery from foot drop.

## Data Availability Statement

The raw data supporting the conclusions of this article will be made available by the authors, without undue reservation.

## Ethics Statement

The animal study was reviewed and approved by the Institutional Animal Care and Use Committee (IACUC), Chang Gung University with IACUC Approval No: CGU111-002.

## Author Contributions

M-YC, C-WK, and T-HH conceived and designed the experiments. S-YC, C-WK, M-YC, and T-HH performed the experiments. T-HH, M-YC, T-TL, and C-WP provided the equipment. M-YC, C-WK, S-YC, C-WP, and T-HH developed the methodology. S-YC, C-WK, and T-HH analyzed the data. S-YC, C-WK, M-YC, T-TL, and T-HH contributed to the writing and editing the manuscript. All authors contributed to the article and approved the submitted version.
